# Italian Validation of the Delaying Gratification Inventory in Adolescents

**DOI:** 10.3390/ijerph20156527

**Published:** 2023-08-05

**Authors:** Ziqin Liang, Elisa Delvecchio, Adriana Lis, Claudia Mazzeschi

**Affiliations:** 1 Department of Philosophy, Social Sciences and Education, University of Perugia, Perugia, Piazza Ermini 1, 06123 Perugia, Italy; 2 Department of Developmental Psychology and Socialization, University of Padova, 06123 Padova, Italy

**Keywords:** delay of gratification, adolescents, psychometric properties, factor structure, self-assessment

## Abstract

The delay of gratification (DoG) is defined as the willingness to forego immediate satisfaction to achieve greater long-term gratification. This ability is essential in adolescence, as its development is crucial against desirable versus undesirable behaviors. This study investigated the psychometric proprieties of the Delaying Gratification Inventory (DGI) in Italian adolescents. A total of 621 Italian adolescents, ranging from 14 to 17 years old (*M* = 15.92, *SD* = 1.05; 47.7% boys), participated in the study. Confirmatory factor analysis identified a four-factor structure (delay of gratification concerning Food, Social Interaction, Money, and Achievement). The reliability of the Italian DGI was acceptable. Measurement invariance across gender was supported. Boys reached a higher DoG score in the Food subscale and a lower score in the Social Interaction and Achievement subscales than girls. Moreover, the Italian DGI dimensions were all positively correlated with self-control and prosocial behavior. Except for the Social Interaction subscale, positive correlations were found between the Food, Money, and Achievement subscales and self-esteem. The present findings suggest that the Italian DGI could be used for assessing DoG ability, a key regulatory ability that promotes healthy behaviors in adolescence.

## 1. Introduction

The delay of gratification (DoG) is defined as the willingness to forego immediate satisfaction to achieve greater long-term gratification and rewards [1]. The DoG process represents the ability to inhibit behavior in the presence of an immediately available stimulus [2]. In other words, the DoG is the ability to make choices by inhibiting impulsive behavior.

The DoG involves multiple positive aspects, such as individual personality and behavioral characteristics [3]. In particular, the DoG is associated with positive personal traits (e.g., self-esteem) and healthy behaviors (e.g., prosocial behavior). Students with higher levels of DoG are better at managing their efforts and choose longer-term academic goals (such as degrees) as rewards [4]. In a series of experiments involving 12- to 18-year-old adolescents, Wulfert et al. [5] found that students who chose immediate rewards showed lower self-esteem than students who chose delayed gratification rewards. Moreover, researchers have argued that prosocial behaviors, including reciprocal interactions, require the DoG, defined as the ability to overcome selfish impulses to bear personal costs in the short term for long-term benefits [6,7]. In childhood, prosocial behaviors are related to the need for achievement [8]. Experimental methods to assess the DoG in childhood have shown that individuals with a high level of DoG show more prosocial behaviors [7,9,10]. Moreover, Hoerger et al. [11] suggested that delayed gratification was an aspect of self-control and self-regulation [12], while self-control can be considered as a continuum of the DoG [11]. Because the implementation of DoG involves choosing between immediate rewards and related long-term effects, the DoG and self-control have been reported to have similar structures and to be interdependent [13,14]. There is also support for the idea of a bidirectional relationship between self-control and the DoG [15].

Adolescence is a particularly critical period for the development of DoG ability. First, in this stage, the development of self-regulatory strategies represents an important task against desired versus undesired behaviors. This period foresees an unbalanced maturation between the higher-order cerebral structures responsible for emotional and behavioral regulation, and the subcortical, emotion-driven structures influencing both emotional and behavioral responses [16]. Findings from developmental neuroscience studies [17] also suggest that adolescence is characterized by increased experimentation with novelty. Because of this unbalance of cerebral maturation and increased wish for experimentation with novelty, adolescents have difficulty controlling impulses and lack the capacity to efficiently allocate attention away from a desired object.

Previous studies have found that adolescents are at greater risk of risky behaviors, such as the use of alcohol, tobacco, and other drugs [17,18], and adjustment problems [16]. However, studies have shown that a higher level of DoG is associated with greater self-control and less impulsivity [17], better working memory and motor inhibition [19], and better academic performance [20]. So, the ability to delay gratification can be considered as a potential source of controlling tendencies towards risky/problematic behaviors as well as promoting healthy behaviors in adolescence. Because adolescence is an important stage for delayed gratification, focusing the attention on a reliable instrument to measure the level of adolescents’ DoG would contribute to assessing this specific ability to achieve long-term gratification and reward in adolescents.

The use of self-report measures has the advantage of being low cost and quick and allowing the recruitment of large samples [11], which can provide a great deal of convenience for assessing adolescents’ DoG. Moreover, when validated in different countries, they could provide a reliable tool for cross-cultural studies [21]. The aim of this study was to validate a self-report measure of the DoG, the Delaying Gratification Inventory (DGI; [11]), in an Italian sample of adolescents by examining some of its psychometric properties.

The DoG involves different domains [11]. However, questionnaires devised to measure the DoG tend to focus on specific aspects (academic, vocational, etc.), such as the Delay-of-gratification Gist Scale [18] and the Academic Delay of Gratification Scale [22]. These instruments focus on a single dimension and are not suitable to support the multiple aspects of the DoG [23].

A comprehensive assessment of the DoG requires a multidimensional questionnaire [23]. For this reason, Hoerger et al. [11] developed their five-factor Delaying Gratification Inventory (DGI), which covers five subscales: Food, Physical Pleasure, Social Interaction, Money, and Achievement. They validated their tool in adult populations in different countries and cultures (including the USA, Canada, South America, Asia, Europe, Mexico, Australia, and Africa) using an online survey. DGI has been translated into multiple languages and used in many countries, such as Poland [14], Brazil [12], Spain [21], and Turkey [24]. The five-factor structure has been confirmed in the adult population (Poland, Brazil, and Turkey) and in adolescents (Spain), and evidence of validity and reliability was provided. However, it should be noted that the internal consistency of some subscales was slightly lower in the previous validation. For example, the Physical Pleasure subscale showed low reliability in the Polish [14], Brazilian [12], Spanish [21], and Turkish [24] validation; the Social Interaction subscale also showed some issues in the Brazilian [12], Spanish [21], and Turkish [24] validation; and the Food subscale showed the same shortcomings in the Polish [14] and Spanish [21] validation. Dymek and Jurek [14] pointed out that this may be attributed to cultural differences. For example, different cultural values have different levels of acceptance of social and physical contact [25]. Thus, verifying the Italian version of DGI would be helpful for further cross-cultural studies. Moreover, although Hoerger et al. [11] included a subsample of adolescents in their DGI validity study, only Espada et al. [21] validated the DGI with adolescents in Spain.

In addition, although some studies have examined gender differences in the DoG, different results have been found. Some studies have found no gender differences in the DoG [5,21]. However, some studies have suggested that gender differences in the DoG appear early in development [26,27]. Moreover, some researchers have found that females consistently displayed a higher level of the DoG than males [11,28]. Others have suggested that gender difference is not a permanent state and that maturation and hormonal changes during adolescence and adulthood affect an individual’s impulsivity, which influences the brain, cognition, and behavior [29]. Girls’ DoG ability seems to fluctuate with hormonal changes due to the menstrual cycle [30]. In contrast, boys, who have higher prenatal testosterone levels, are not able to delay gratification as easily [21]. However, all these mixed results could be easily affected by the accuracy of the measuring instruments used, the different samples examined, and the sample size [28]. In terms of the DGI, Hoerger et al. [11] found that females scored higher than males on the Physical Pleasure, Social Interaction, and Achievement subscales, while males had a greater advantage in delayed gratification in the Food subscale. However, Espada et al. [21] did not find gender differences in all the subscales of the DGI in a sample of Spanish adolescents. Considering the inhomogeneity of these results, also concerning the DGI, it would be important to explore adolescents’ gender differences in the subscales of the Italian version of the DGI when administered to adolescents.

Furthermore, in terms of the relations between the DGI and other positive variables, although no studies have directly correlated the DoG with self-esteem, relevant studies have shown a strong relationship between them [5,31]. And Hoerger et al. [8] found that the DGI was positively correlated with prosocial behavior, particularly in the Social Interaction dimension. Therefore, using the DGI to assess the DoG would help explore the role of the DoG in positive personal traits and healthy behaviors in adolescents.

The current study aimed to assess the psychometric properties of the DGI in a sample of Italian adolescents. Specifically, this study aimed to perform the following objectives: (1) examine the internal consistency of the Italian DGI; (2) test the factor structure of the Italian DGI [11,21]; (3) test its measurement invariance across genders; (4) assess if boys have higher levels of the DoG than girls on the Food subscale, but lower levels of the DoG on Physical Pleasure, Social Interaction, and Achievement subscales [11]; and (5) examine correlations between the Italian DGI subscales and self-control [11,14,21,32], self-esteem [5], and prosocial behaviors [11].

## 2. Materials and Methods

### 2.1. Participants

The sample size was determined by Soper’s [33] a priori sample size calculator for structural equation models. Based on a medium effect size (i.e., 0.30) with 0.80 as the desired statistical power and 0.05 as the probability level, the results indicated that the recommended minimum sample size should be 400 for a model with 5 latent and 35 observed variables.

The sample included 621 Italian adolescents ranging from 14 to 17 years old (*M* = 15.92 years, *SD* = 1.05; 47.7% boys). The participants were recruited from high schools located in Northern Italy through a convenience sampling method as volunteers, primarily serving working and middle-class families, in urban and suburban school districts. Age skewness was 0.39 and kurtosis was −0.81, so age distribution can be considered normal. In detail, 82 adolescents were 14-years-old, 121 were 15-years-old, 184 were 16-years-old, and 234 were 17-years-old. More than 90% of the participants came from two-parent households. All participants indicated they had never been hospitalized because of psychiatric symptoms in the past two years. A few (<5% of the total sample) reported previous psychological counseling or intervention in the past two years for mild problems, such as sleep problems as well as short-term emotional upheaval. Thus, no participants were excluded due to psychiatry history information.

### 2.2. Procedures

The administration was carried out according to the ethical standards for research outlined in the Code of Ethics of the World Medical Association (Declaration of Helsinki). This study was approved by the Ethical Committee for psychological research of the author’s university. School approval and parents’ signed consent were obtained before data collection, and all the students involved provided consent before participation. Voluntary participation and anonymity were highlighted. The anonymous questionnaires were numbered after they were collected to ensure confidentiality. Trained master’s students majoring in psychology and who were familiar with the questionnaires administered the measures to the students during regular school hours. Participants were asked to be open and honest and to refrain from sharing their answers with others. The participants completed paper and pencil questionnaires, which took approximately 30 min.

### 2.3. Measures

#### 2.3.1. The Delaying Gratification Inventory

The Delaying Gratification Inventory (DGI; [11]) is a self-report measure composed of 35 items to evaluate the ability of delayed gratification in five dimensions (subscales of Food, Physical Pleasure, Social Interaction, Money, and Achievement). Responses are scored on a 5-point Likert scale (from “1 = strongly disagree” to “5 = strongly agree”). Higher scores indicate a better capacity for self-regulation to delay immediate gratification and achieve greater long-term gratification. With the copyright holder’s permission, the Italian translation of the DGI-35 was developed following the guidelines of Muñiz et al. [34] and the International Test Commission [35]. Sample items are “I can resist junk food when I want to” and “I try to spend my money wisely”.

#### 2.3.2. Self-Control

The Brief Self-Control Scale (BSCS; [36]) was used to assess participants’ ability to control their impulses, alter their emotions and thoughts, interrupt undesired behavioral tendencies, and refrain from acting on them. This scale has 13 items rated on a five-point scale (from “1 = not like me at all” to “5 = very much like me”), with a higher score indicating better self-control ability. Sample items are “I am good at resisting temptation” and “It is hard for me to get rid of bad habits”. The scale has been used in Italian adolescents and shown good psychometric properties [37]. In the present study, the ordinal alpha for BSCS was 0.76 (95% CI = [0.74, 0.79]).

#### 2.3.3. Self-Esteem

The Rosenberg Self-Esteem Scale (RSES; [38]) consists of 10 items rated on a 4-point scale (from “1 = strongly disagree” to “4 = strongly agree”). Higher scores indicate higher self-esteem. Sample items are “On the whole, I am satisfied with myself” and “All in all, I am inclined to think that I am a failure”. The Italian versions of this scale have been validated and used, showing good psychometric properties [39]. In the present study, the ordinal alpha for RSES was 0.87 (95% CI = [0.86, 0.89]).

#### 2.3.4. Prosocial Behavior

The Strengths and Difficulties Questionnaire (SDQ; [40]) is a brief screening tool to assess strengths (prosocial behavior) and adjustment difficulties (a total difficulty score including four difficulty subscales). The Prosocial Behavior subscale consists of 5 items rated on a 3-point scale (from “0 = not true” to “2 = certainly true”). Prosocial behavior assesses resources rather than problems. Prosocial resources are related to social skills or competences and adaptive behavior. Prosocial behavior is not included in the total difficulty score because a lack of prosocial behavior problems is conceptually different from the presence of psychological difficulties [41]. Higher scores indicate more prosocial behavior. Sample items are “I usually share with others (food, games, pens etc.)” and “I often volunteer to help others (parents, teachers, children)”. In the present study, the ordinal alpha for the Prosocial Behavior subscale was 0.87 (95% CI = [0.86, 0.89]).

### 2.4. Data Analysis

First, some preliminary analyses were carried out. Descriptive analyses were run for each item of the DGI (see Table S1 in the Supplementary Materials). Then, interitem and item–rest polychoric correlations were calculated for the five subscales devised by Hoerger et al. [11]. Due to the ordinal Likert-type items and the multivariate non-normality (Mardia’s test of multivariate kurtosis = 51.35, *p* < 0.001) of the data, a polychoric correlation matrix was considered as more appropriate than a Pearson correlation matrix [42], which was used to assess the relations between scores on items of each DGI subscale. The internal consistency for the subscales was evaluated using the ordinal alpha [42]. An alpha value ≥ 0.70 was considered sufficiently reliable, whereas an alpha value ≥ 0.80 was considered good [42]. The diagonally weighted least squares (DWLS) method with robust standard errors and a scaled–shifted test statistic was selected to perform a confirmatory factor analysis (CFA) to test the factorial validity [43]. Values of the root mean square error of approximation (RMSEA) < 0.07 [44], comparative fit index (CFI), Tucker–Lewis index (TLI) > 0.90 [45], and standardized root mean square residual (SRMR) < 0.08 [46] indicated the model fit was acceptable. Next, according to Wu and Estabrook [47] as well as Svetina et al. [48], we used multigroup CFAs to evaluate measurement invariance across genders. Configural, threshold, metric (loadings), and scalar (intercepts) invariance were tested and using delta parameterization. The cut-off values followed Chen’s [49] suggestions, with –0.010 for ΔCFI, 0.010 for ΔTLI, 0.015 for ΔRMSEA, and 0.030 (for metric invariance) or 0.010 (for scalar invariance) for ΔSRMR. If scalar invariance was established, we analyzed latent mean differences across genders [50]. The “boys group” served as the reference group, with the latent mean constrained to zero and freely estimated for girls. In this case, the latent mean of the other group represents the difference between the latent means of the two groups. According to Cohen [51], *d* values between 0.20 and 0.49 are small, between 0.50 and 0.79 are medium, and above 0.80 are large. Finally, to further test the validity of the DGI, Spearman correlations were calculated to analyze the relations between the DGI subscales and self-control (BSCS) and self-esteem (RSES). Correlation effect sizes were interpreted according to Cohen [52], with correlation coefficients of 0.10, 0.30, and 0.50 representing small, medium, and large effect sizes, respectively.

All statistical analyses were performed using the JASP (Version 0.16) and R (Version 4.0.5). JASP was used for descriptive analyses and Spearman correlations. In R, the *psych* package [53] was used for polychoric correlations, the *ufs* package [54] was used for reliability, and the *lavaan* [55] and *semTools* [56] packages were used for CFA and multigroup CFAs.

## 3. Results

### 3.1. Preliminary Analyses

As for item interrelations among the five subscales, the Food, Social Interaction, Money, and Achievement subscales showed the same trend. For each of these four subscales, items were significantly intercorrelated (average interitem polychoric correlation: Food: 0.24; Social Interaction: 0.34; Money: 0.43; and Achievement: 0.41), and the item–rest polychoric correlations were mostly higher than the criterion of 0.3 [57]. In contrast, for the Physical Pleasure subscale, the trend was different due to the fact that the interitem correlations were almost all not significant (the average interitem polychoric correlation was 0.09) and almost all item–rest polychoric correlations were lower than 0.3. These results are shown in the Supplementary Materials (Tables S2–S6).

Adequate reliability was found for the DGI Food, Social Interaction, Money, and Achievement subscales; the ordinal alpha ranged from 0.68 to 0.84 (Food: α = 0.68 [0.65, 0.72]; Social Interaction: α = 0.78 [0.76, 0.81]; Money: α = 0.84 [0.82, 0.86]; and Achievement: α = 0.83 [0.81, 0.85]). However, as expected from the described items’ interrelations, the Physical Pleasure subscale showed a very low value: α = 0.42, 95% CI = [0.35, 0.49].

### 3.2. Factor Validity

The results concerning the Physical Pleasure subscale showed many shortcomings concerning the ability of its items to adequately measure a reliable construct. This could influence the five-factor structure of the DGI, and for this reason, a four-factor structure with the deletion of the scale should also be considered [57,58].

For these reasons, in order to evaluate the factor structure of DGI, two different models were tested: (a) the five-factor original model, which was proposed by the original authors [11] and confirmed by Espada et al. [21] in a sample of Spanish adolescents; (b) a four-factor model, with item loadings on Food, Social Interaction, Money, and Achievement (excluding physical Pleasure).

**Figure 1:**
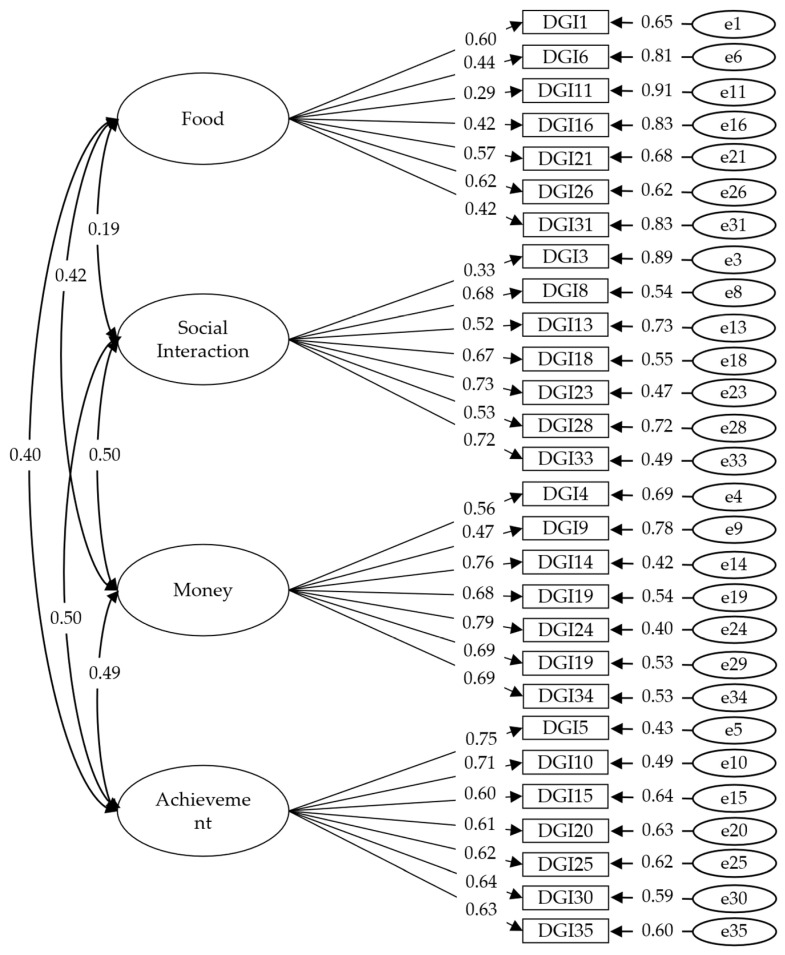
Standardized factor loadings of the four-factor model for the Italian Delaying Gratification Inventory. Note: DGI1–DGI35 = Item 1–Item 35 of the Delaying Gratification Inventory.

The CFA’s results are provided in Table 1; the CFI and TLI values for the five-factor model did not meet the fitting criterion, whereas all the goodness-of-fit indices for the four-factor model were acceptable. Thus, the four-factor model was considered more adequate in the current sample. The standardized factor loadings for the four-factor model are shown in Figure 1 (the “Std.all” column in the *lavaan* package).

### 3.3. Measurement Invariance across Gender

As shown in Table 2, the MCFA results on the DGI four-factor model confirmed configural, threshold, metric, and scalar invariance across genders, suggesting that the factor structure of the DGI was invariant for boys and girls.

### 3.4. Gender Differences

Given the support for scalar invariance across genders, we conducted a comparison of latent mean differences. A chi-squared difference test showed significant latent mean differences: Δχ2 = 75.59 and ∆*df* = 8, *p* < 0.001. Specifically, compared to girls, boys had higher mean scores in the Food subscale, but lower mean scores in the Social Interaction and Achievement subscales (Table 3). No significant gender difference in the Money subscale was found.

### 3.5. Correlations between DGI Subscales, Self-Control, Self-Esteem, and Prosocial Behavior

As shown in Table 4, all the subscales (i.e., Food, Social Interaction, Money, and Achievement) of the DGI were significantly and positively correlated with BSCS, with medium-to-high effect sizes. Except the Social Interaction subscale, correlations between the Food, Money, and Achievement subscales and the RSES were significant and positive, and had small-to-medium effect sizes. All the subscales of the DGI were significantly and positively correlated with prosocial behavior, with small effect sizes.

## 4. Discussion

This study aimed to provide evidence for the psychometric properties of the DGI in a sample of Italian adolescents. The current study supported the four-factor model for the Italian DGI. The reliabilities of the Food, Social Interaction, Money, and Achievement subscales, measurement invariance across genders, and the validity were also supported by the results.

In the current study, Social Interaction, Money, and Achievement showed adequate reliability, in line with previous studies [12,14,21,24]. Although the internal consistency of the Food subscale was slightly lower than the acceptable critical value of the rule-of-thumb, it was similar to the reported data in the Spanish [21] and Polish [14] validation. However, the Physical Pleasure subscale clearly showed some shortcomings in the inter-item and item–rest correlations. As a result, the reliability of this subscale was very low and unacceptable, which was also present in Polish [14], Brazilian [12], and Spanish studies [21]. Espada et al. [21] (2019) evidenced some problems with this subscale in adolescents and suggested that this “may be due to the attempt to maintain the essence of items originally designed for the adult population. It might be advisable to re-word the items according to characteristics and lifestyles typical of adolescence” (p. 332). In terms of the content of the items involved in the Physical Pleasure subscale, a qualitative analysis can show how the subscale includes different aspects of physical pleasure. Some items just refer to control “physical desires” (e.g., item 2: “I am able to control my physical desires”, and item 17: “I have given up physical pleasure or comfort to reach my goals”) and only two specific items refer clearly to specific issues (item 7: “I like to get to know someone before having a physical relationship” and item 22: “I prefer to explore the physical side of romantic involvements right away”).

On the one hand, maybe the term “physical desires” is possibly vague for adolescents who are facing all the complex issues of sexuality, of new body experiences, of romantic relationships, and so on. On the other hand, concerning the specific items that evaluate physical versus romantic relationships, it could be difficult to answer for Italian adolescents. Italy is a country with strong family ties, Catholic values, and a slow transition to adulthood; studies have shown that this kind of Italian culture influences the sexual initiation of young Italians, which tends to be slow [59]. In contrast to previous studies, the original five-factor model [11,21] was not supported. The fragility of the Physical Pleasure subscale looked likely to influence the structural validity of the DGI. In fact, the results of the CFA supported the four-factor structural model (i.e., Food, Social Interaction, Money, and Achievement) of the Italian DGI with good fit indices. The authors acknowledged the importance of the Physical Pleasure scale and were not satisfied with the decision to remove it. However, preliminary analyses aimed to retain the scale; even substantially reducing its number of items did not show encouraging results. The results of the CFA, as well as the trends in intercorrelations and item–rest correlations, made it difficult to find at least some items to form a subscale, due to the fact that they were low or not significant.

It is very important to underline how basic these dimensions are for a mature DoG in adolescence. Furthermore, gender invariance was established, suggesting that the four-factor structure of the Italian DGI is applicable to both boys and girls in adolescence.

However, some gender differences were found in the Italian DGI. The Social Interaction and Achievement subscales were higher in girls, whereas boys showed a higher level of the DoG in the Food subscale. No significant gender differences were found for the Money subscale. These results are similar to the findings of Hoerger et al. [11]. In adolescence, girls gradually grow up and become more independent, and compared with boys, they have a higher ability for self-discipline and self-regulation [11,28]. The Social Interaction subscale is related to altruistic and prosocial behaviors [11]. Previous evidence supports the idea that girls take on more responsibility than boys in interpersonal communication [60]. The Achievement subscale is related to conscientiousness and striving for achievement [11]. To obtain the benefits of interpersonal communication and learning achievements [20], girls need to suppress short-term temptation to achieve long-term goals and so are more likely to show a higher level of ability for delayed gratification. Interestingly, in terms of the Food subscale, boys had higher delayed gratification than girls. Previous studies have shown that disordered eating behaviors (e.g., binge eating and loss of control eating) are already common in adolescence [61] and the prevalence of eating disorder features is significantly higher in girls than in boys [62,63]. These behaviors are generally related to executive functioning (e.g., inhibition and delayed gratification) [64]. Our results suggest the importance of delayed gratification in food in relation to problems regulating eating behaviors and obesity-related behaviors. There was no gender difference in the Money subscale, probably because almost all adolescents are dependent on family support [65]. Moreover, compared to the economic world of adults, adolescents are not fully contractually capable yet [66]. Thus, their spending opportunities are still restricted.

Finally, this study examined the validity of the Italian DGI by testing the correlation between the Italian DGI and self-control and self-esteem. The results showed that self-control was significantly and positively related to four subscales of the Italian DGI, which is consistent with previous findings [11,14,21,32]. It confirms the close relation between self-control and delayed gratification. Adolescents with higher levels of delayed gratification have a strong ability to self-control, which can be considered an operational indicator of impulse control [2,11]. As for the four subscales of the Italian DGI and self-esteem, significant correlations were found for all subscales, except Social Interaction. However, the effect size was low. This seems to suggest that self-esteem plays some role in the DoG, but other intervening variables need to be considered in this relation. The Social Interaction subscale of the DGI underlines the ability to respond to the needs and perspective of others in adolescents. Self-esteem, on the other hand, involves individuals’ subjective judgment to their ability to value themselves [38]. That is, the two abilities are related to the motivation towards others and the motivation towards oneself, respectively, which may explain the insignificant correlation between the Social Interaction subscale and self-esteem. In addition, there were positive correlations between the four subscales of the Italian DGI and prosocial behavior, though with small effect sizes. These results are also in line with previous studies [6,7,9,11], suggesting the positive relations between the DoG and prosocial behavior, and providing evidence that the DoG correlates with prosocial behavior in multiple dimensions. Previous studies have suggested that the relation between the DoG and prosocial behavior may be influenced by other factors, such as the type of prosocial behavior [7]. Altogether, these relations provide further evidence for the validity of the Italian version of the DGI.

However, the present study had some limitations. First, as it is a cross-sectional study, it would be difficult to determine whether the delayed gratification ability of adolescents will change with age. Future longitudinal studies are needed to explore the suitability of this instrument for different ages. Second, the convenience sampling method was used in this study, which may lead to biased estimates of the overall population. Since the sample was not chosen through random selection, it may not be fully representative of the population being studied. This undermines the ability to make generalizations from this sample to the population of interest. Moreover, the data referred to a single data source (self-reported), and most were collected from Northern Italy. These may hinder the generalization of the results. Future research should collect data from adolescents across all the regions of Italy. Third, due to the unsatisfactory results of the Physical Pleasure subscale in the Italian version, this subscale was regrettably removed from the present study. It is strongly recommended that future studies could explore the delay of gratification of physical pleasure in depth and develop a scale for it, which would meaningfully and more accurately measure this dimension of delay of gratification. Fourth, it would be fruitful for future studies to investigate other important variables and the mechanisms underlying the deficits and/or strengths of the DoG, such as internalizing and externalizing problems. This would help to detect and identify relevant variables in different dimensions of DoG contexts in adolescents, in order to support the development of healthy behaviors and prevent the occurrence of problematic and risky behaviors.

## 5. Conclusions

The current study was the first validation of the Italian DGI, confirming the adequacy of the four-factor structure model in Italian adolescents and establishing its measurement invariance across genders. The internal consistency of its subscales was acceptable. Validity was assessed by examining the correlations of the DGI with self-control, self-esteem, and prosocial behavior, with significant correlations delivered. The Italian DGI will aid our understanding and detection of deficits in the DoG ability of adolescents. Further exploration of its clinical application value will help to reduce the risk of problem behaviors and lead to a better understanding of the development of adolescents. In general, this scale can be used as a reliable tool for assessing the DoG in Italian adolescents.

## Figures and Tables

**Table 1:** Results of the confirmatory factor analysis.

Models	χ2	*df*	*p*	CFI	TLI	RMSEA (90% CI)	SRMR
M1: five-factor	1472.61	550	<0.001	0.896	0.888	0.052 (0.049, 0.055)	0.063
M2: four-factor	947.363	344	<0.001	0.925	0.917	0.053 (0.049, 0.057)	0.059

Note: *N* = 621; CFI = comparative fit index; TLI = Tucker–Lewis index; RMSEA = root mean square error of approximation; SRMR = standardized root mean square residual.

**Table 2:** Measurement invariance of the Italian DGI across genders.

	χ2	*df*	Δχ2	RMSEA	ΔRMSEA	CFI	ΔCFI	TLI	ΔTLI	SRMR	ΔSRMR
Boys (*n* = 296)	623.31	344		0.052		0.917		0.909		0.068	
Girls (*n* = 325)	646.80	344		0.052		0.924		0.917		0.069	
Configural	1295.0	688		0.052		0.921		0.913		0.069	
Threshold	1317.1	744	48.86	0.051	−0.002	0.920	−0.001	0.919	0.005	0.069	0.000
Metric	1405.6	768	39.58 *	0.050	−0.001	0.921	0.001	0.922	0.003	0.070	0.001
Scalar	1543.7	792	88.05 ***	0.051	0.002	0.913	−0.007	0.917	−0.005	0.070	0.000

Note: CFI = comparative fit index; TLI = Tucker–Lewis index; RMSEA = root mean square error of approximation; SRMR = standardized root mean square residual; Δ = change between model and comparison model in the row above; * *p* < 0.05, *** *p* < 0.001.

**Table 3:** Latent mean differences across genders.

	Boys (*n* = 296)		Girls (*n* = 325)		*z*	*p*	*d*
	*M*	*SD*	*M*	*SD*			
Food	0.08	0.04	−0.08	0.05	2.63	0.008	0.26
Social Interaction	−0.09	0.03	0.10	0.04	−4.40	<0.001	−0.41
Money	−0.03	0.04	0.04	0.05	−1.11	0.266	−0.10
Achievement	−0.19	0.05	0.20	0.05	−5.53	<0.001	−0.50

**Table 4:** Spearman correlations between Italian DGI subscales and other variables.

	Food	Social Interaction	Money	Achievement
BSCS	0.39 ***	0.23 ***	0.40 ***	0.49 ***
RSES	0.21 ***	0.06	0.13 ***	0.28 ***
PRO	0.15 ***	0.20 ***	0.18 ***	0.15 ***

Note: BSCS = the Brief Self-Control Scale; RSES = Rosenberg’s Self-Esteem Scale; PRO = the Strengths and Difficulties Questionnaire—the Prosocial Behavior subscale; *** *p* < 0.001.

## Data Availability

The data presented in this study are available on request from the corresponding author.

## Supplementary Materials

The following supporting information can be downloaded at: https://www.mdpi.com/article/10.3390/ijerph20156527/s1. Table S1: Descriptive statistics for the Italian Delaying Gratification Inventory (DGI); Table S2: Polychoric correlations between Italian DGI Food subscale items; Table S3: Polychoric correlations between Italian DGI Social Interaction subscale items; Table S4: Polychoric correlations between Italian DGI Money subscale items; Table S5: Polychoric correlations between Italian DGI Achievement subscale items; Table S6: Polychoric correlations between Italian DGI Physical Pleasure subscale items.
