# Supplementary material for: Italian Validation of the Delaying Gratification Inventory in Adolescents
Source: Int J Environ Res Public Health. 2023 Aug 5;20(15):6527. doi: 10.3390/ijerph20156527 (PMC10419053; doi:10.3390/ijerph20156527)
Supplement: Supplementary file 1 [file ijerph-20-06527-s001.zip › ijerph-2379957-supplementary.pdf]

Supplementary Material

Table S1. Descriptive statistics for the Italian Delaying Gratification Inventory (DGI).

Factor	Item	*M*	*SD*	Skewness	Kurtosis
Food	DGI1. I can resist junk food when I want to.	3.66	1.27	−0.55	−0.86
	DGI6. I would have a hard time sticking with a special, healthy diet. (R)	2.80	1.32	0.17	−1.04
	DGI11. If my favorite food were in front of me, I would have a difficult time waiting to eat it. (R)	3.65	1.25	−0.61	−0.65
	DGI16. It is easy for me to resist candy and bowls of snack foods.	3.02	1.24	0.02	−0.99
	DGI21. Sometimes I eat until I make myself sick. (R)	2.20	1.30	0.68	−0.86
	DGI26. I have always tried to eat healthy because it pays off in the long run.	2.99	1.13	0.04	−0.64
	DGI31. Even if I am hungry, I can wait until it is meal time before eating something.	3.08	1.17	−0.02	−0.91
Physical Pleasure	DGI2. I am able to control my physical desires.	3.75	0.97	−0.48	−0.22
	DGI7. I like to get to know someone before having a physical relationship.	4.19	1.10	−1.34	0.98
	DGI12. My habit of focusing on what "feels good" has cost me in the long run. (R)	3.11	1.06	−0.15	−0.36
	DGI17. I have given up physical pleasure or comfort to reach my goals.	2.82	1.05	−0.01	−0.31
	DGI22. I prefer to explore the physical side of romantic involvements right away. (R)	2.43	1.16	0.35	−0.79
	DGI27. When faced with a physically demanding chore, I always tried to put off doing it. (R)	2.45	1.09	0.41	−0.49
	DGI32. I have lied or made excuses in order to go do something more	3.12	1.09	−0.19	−0.56

	pleasurable. (R)				
Social Interaction	DGI3. I hate having to take turns with other people. (R)	2.83	1.13	0.08	−0.56
	DGI8. Usually I try to consider how my actions affect others.	3.65	0.95	−0.60	0.24
	DGI13. I think that helping each other benefits society.	4.20	0.90	−1.23	1.55
	DGI18. I try to consider how my actions will affect other people in the long-term.	3.49	0.99	−0.54	0.08
	DGI23. I do not consider how my behavior affects other people. (R)	2.15	1.02	0.60	−0.22
	DGI28. I value the needs of other people around me.	3.53	0.91	−0.48	0.11
	DGI33. There is no point in considering how my decisions affect other people. (R)	2.18	1.02	0.49	−0.52
Money	DGI4. When I am able to, I try to save away a little money in case an emergency should arise.	3.57	1.26	−0.52	−0.83
	DGI9. It is hard for me to resist buying things I cannot afford. (R)	2.52	1.20	0.43	−0.75
	DGI14. I try to spend my money wisely.	3.69	1.08	−0.59	−0.27
	DGI19. I cannot be trusted with money. (R)	1.90	1.09	1.15	0.65
	DGI24. When someone gives me money, I prefer to spend it right away. (R)	1.99	1.08	0.99	0.38
	DGI29. I manage my money well.	3.64	1.05	−0.59	−0.14
	DGI34. I enjoy spending money the moment I get it. (R)	2.41	1.15	0.46	−0.63
Achievement	DGI5. I worked hard in school to improve myself as a person.	3.31	1.12	−0.22	−0.66
	DGI10. I have tried to work hard in school so that I could have a better future.	3.50	1.11	−0.40	−0.50
	DGI15. In school, I tried to take the easy way out. (R)	2.77	1.22	0.23	−0.85
	DGI20. I am capable of working hard to get ahead in life.	3.75	0.98	−0.69	0.21
	DGI25. I cannot motivate myself to accomplish long-term goals. (R)	2.36	1.13	0.35	−0.71

	DGI30. I have always felt like my hard work would pay off in the end.	3.73	1.05	−0.56	−0.26
	DGI35. I would rather take the easy road in life than get ahead. (R)	2.15	1.02	0.60	−0.23

Note: The "(R)" at the end of the item indicates this item should be reversely scored in calculating total score.

Table S2. Polychoric correlations between Italian DGI Food subscale items.

	**1**	**2**	**3**	**4**	**5**	**6**	**7**
1. DGI1	—						
2. DGI6 (R)	0.26	—					
3. DGI11 (R)	0.28	0.28	—				
4. DGI16	0.24	0.16	0.21	—			
5. DGI21 (R)	0.25	0.18	0.16	0.17	—		
6. DGI26	0.31	0.40	0.18	0.37	0.23	—	
7. DGI31	0.23	0.25	0.21	0.13	0.22	0.22	—
8. Item-rest correlation	0.44	0.43	0.36	0.35	0.33	0.48	0.34

Note: The "(R)" at the end of the item indicates this item was reversely scored.

Table S3. Polychoric correlations between Italian DGI Social Interaction subscale items.

	**1**	**2**	**3**	**4**	**5**	**6**	**7**
1. DGI3 (R)	—						
2. DGI8	0.23	—					
3. DGI13	0.14	0.28	—				
4. DGI18	0.18	0.58	0.28	—			
5. DGI23 (R)	0.27	0.46	0.29	0.49	—		
6. DGI28	0.15	0.36	0.36	0.35	0.34	—	
7. DGI33 (R)	0.21	0.45	0.33	0.45	0.54	0.41	—
8. Item-rest correlation	0.28	0.60	0.41	0.59	0.61	0.49	0.61

Note: The "(R)" at the end of the item indicates this item was reversely scored.

Table S4. Polychoric correlations between Italian DGI Money subscale items.

	**1**	**2**	**3**	**4**	**5**	**6**	**7**
1. DGI4	—						
2. DGI9 (R)	0.19	—					
3. DGI14	0.48	0.38	—				
4. DGI19 (R)	0.33	0.39	0.46	—			
5. DGI24 (R)	0.47	0.34	0.52	0.50	—		
6. DGI29	0.44	0.30	0.58	0.47	0.52	—	
7. DGI34 (R)	0.43	0.33	0.46	0.41	0.65	0.42	—
8. Item-rest correlation	0.53	0.43	0.67	0.59	0.70	0.63	0.63

Note: The "(R)" at the end of the item indicates this item was reversely scored.

Table S5. Polychoric correlations between Italian DGI Achievement subscale items.

	**1**	**2**	**3**	**4**	**5**	**6**	**7**
1. DGI5	—						
2. DGI10	0.67	—					
3. DGI15 (R)	0.49	0.49	—				
4. DGI20	0.42	0.42	0.28	—			
5. DGI25 (R)	0.33	0.37	0.32	0.36	—		
6. DGI30	0.41	0.41	0.29	0.48	0.43	—	
7. DGI35 (R)	0.37	0.32	0.41	0.37	0.44	0.43	—
8. Item-rest correlation	0.64	0.64	0.53	0.55	0.52	0.58	0.55

Note: The "(R)" at the end of the item indicates this item was reversely scored.

Table S6. Polychoric correlations between Italian DGI Physical Pleasure subscale items.

	1	2	3	4	5	6	7
1. DGI2	—						
2. DGI7	0.18	—					
3. DGI12 (R)	0.12	0.04	—				
4. DGI17	0.09	0.16	−0.10	—			
5. DGI22 (R)	0.14	0.46	0.07	0.07	—		
6. DGI27 (R)	0.08	0.10	0.07	−0.06	−0.03	—	
7. DGI32 (R)	0.05	0.12	0.09	0.02	0.21	0.06	—
8. Item-rest correlation	0.23	0.38	0.09	0.06	0.32	0.0	0.19

Note: The “(R)” at the end of the item indicates this item was reversely scored.

DGI-35

(traduzione a cura di A. Lis e E. Delvecchio)

Legga attentamente ciascuna affermazione e indichi quanto accuratamente ciascun item la descrive segnando con una crocetta (X) il numero appropriato per ogni affermazione. Non impieghi troppo tempo per ogni affermazione e risponda velocemente e onestamente secondo la seguente scala:

1------------------------2------------------------3----------------------4---------------------5

Per niente d'accordo	Né d'accordo né in disaccordo	Molto d'accordo

1. Se lo voglio, posso resistere al cibo spazzatura	1	2	3	4	5
2. Sono capace di controllare i miei bisogni fisici	1	2	3	4	5
3. Odio dover fare a turno con altre persone	1	2	3	4	5
4. Quando mi è possibile, cerco di mettere via un po' di denaro nel caso in cui possa verificarsi qualche emergenza	1	2	3	4	5
5. Lavoro molto duramente a scuola per migliorarmi come persona	1	2	3	4	5
6. Faccio molta fatica ad aderire a una dieta particolare e salutare	1	2	3	4	5
7. Mi piace conoscere qualcuno/a prima di avere con lui/lei una relazione fisica	1	2	3	4	5
8. Di solito cerco di tenere in considerazione come le mie azioni possono influenzare gli altri	1	2	3	4	5
9. Anche se non posso permettermi delle cose, mi è difficile resistere a comperarle	1	2	3	4	5
10. Ho cercato di lavorare duramente a scuola per potere avere un futuro migliore	1	2	3	4	5
11. Se avessi davanti a me il mio cibo favorito mi sarebbe difficile attendere a mangiarlo	1	2	3	4	5
12. La mia abitudine di focalizzarmi su ciò che "mi fa sentire bene", ha avuto delle conseguenze a lungo andare	1	2	3	4	5
13. Credo che aiutarsi uno con l'altro rappresenti un beneficio per la società	1	2	3	4	5
14. Cerco di spendere il mio denaro in modo saggio	1	2	3	4	5
15. A scuola ho sempre cercato di cavarmela nel modo più facile o con delle scappatoie	1	2	3	4	5
16. Mi risulta facile resistere a dolci e/o snack anche in grandi quantità	1	2	3	4	5
17. Ho rinunciato al conforto o al piacere fisico per raggiungere il mio scopo	1	2	3	4	5

18. Cerco di tenere in considerazione come le mie azioni, a lungo termine, potrebbero influire sugli altri	1	2	3	4	5
19. Per quanto riguarda il denaro non c'è da fidarsi di me	1	2	3	4	5
20. Sono capace di lavorare duramente per farmi strada nella vita	1	2	3	4	5
21. Qualche volta mangio fino a sentirmi male	1	2	3	4	5
22. Preferisco esplorare immediatamente l'aspetto fisico del coinvolgimento romantico	1	2	3	4	5
23. Non tengo in considerazione come il mio comportamento possa influenzare gli altri	1	2	3	4	5
24. Quando qualcuno mi dà del denaro preferisco spenderlo immediatamente	1	2	3	4	5
25. Non riesco a motivarmi per raggiungere obiettivi a lungo termine	1	2	3	4	5
26. Ho sempre cercato di mangiare sano perché questo ti ripaga a lungo andare	1	2	3	4	5
27. Quando mi confronto con incombenze che richiedono un impegno fisico, cerco subito di rimandarle	1	2	3	4	5
28. Tengo molto in considerazione i bisogni delle persone che mi circondano	1	2	3	4	5
29. So gestire bene il mio denaro	1	2	3	4	5
30. Ho sempre creduto che il mio duro lavoro alla fine mi ripagherà	1	2	3	4	5
31. Anche se ho fame, posso aspettare fino al momento del pasto prima di mangiare qualcosa	1	2	3	4	5
32. Ho mentito o mi sono scusato per ottenere qualcosa di più piacevole	1	2	3	4	5
33. Non vale la pena pensare a come le mie decisioni potrebbero influenzare gli altri	1	2	3	4	5
34. Mi piace spendere il denaro nel momento in cui ce l'ho	1	2	3	4	5
35. Preferisco intraprendere una strada facile, piuttosto che farmi strada nella vita	1	2	3	4	5

Note: items in red refer to Physical Pleasure
